# Immunoinformatics Approach for Multiepitope Vaccine Prediction from H, M, F, and N Proteins of Peste des Petits Ruminants Virus

**DOI:** 10.1155/2019/6124030

**Published:** 2019-10-30

**Authors:** Bothina B. M. Gaafar, Sumaia A. Ali, Khoubieb Ali Abd-elrahman, Yassir A. Almofti

**Affiliations:** ^1^Department of Molecular Biology and Bioinformatics, College of Veterinary Medicine, University of Bahri, Khartoum, Sudan; ^2^Department of Veterinary Medicine and Surgery, College of Veterinary Medicine, Sudan University of Science and Technology, Sudan; ^3^Department of Pharmaceutical Technology, College of Pharmacy, University of Medical Science and Technology (UMST), Khartoum, Sudan

## Abstract

**Background:**

Small ruminant morbillivirus or peste des petits ruminants virus (PPRV) is an acute and highly contagious viral disease of goats, sheep, and other livestock. This study aimed at predicting an effective multiepitope vaccine against PPRV from the immunogenic proteins haemagglutinin (H), matrix (M), fusion (F), and nucleoprotein (N) using immunoinformatics tools.

**Materials and Methods:**

The sequences of the immunogenic proteins were retrieved from GenBank of the National Center for Biotechnology Information (NCBI). BioEdit software was used to align each protein from the retrieved sequences for conservancy. Immune Epitope Database (IEDB) analysis resources were used to predict B and T cell epitopes. For B cells, the criteria for electing epitopes depend on the epitope linearity, surface accessibility, and antigenicity.

**Results:**

Nine epitopes from the H protein, eight epitopes from the M protein, and ten epitopes from each of the F and N proteins were predicted as linear epitopes. The surface accessibility method proposed seven surface epitopes from each of the H and F proteins in addition to six and four epitopes from the M and N proteins, respectively. For antigenicity, only two epitopes *_142_PPERV_146_* and *_63_DPLSP_67_* were predicted as antigenic from H and M, respectively. For T cells, MHC-I binding prediction tools showed multiple epitopes that interacted strongly with BoLA alleles. For instance, the epitope *_45_MFLSLIGLL_53_* from the H protein interacted with four BoLA alleles, while *_276_FKKILCYPL_284_* predicted from the M protein interacted with two alleles. Although F and N proteins demonstrated no favorable interaction with B cells, they strongly interacted with T cells. For instance, *_358_STKSCARTL_366_* from the F protein interacted with five alleles, followed by *_340_SQNALYPMS_348_* and *_442_IDLGPAISL_450_* that interacted with three alleles each. The epitopes from the N protein displayed strong interaction with BoLA alleles such as *_490_RSAEALFRL_498_* that interacted with five alleles, followed by two epitopes _2_*ATLLKSLAL*_10_ and *_304_QQLGEVAPY_312_* that interacted with four alleles each. In addition to that, four epitopes *_3_TLLKSLALF_11_*, *_356_YFDPAYFRL_364_*, *_360_AYFRLGQEM_368_*, and *_412_PRQAQVSFL_420_* interacted with three alleles each.

**Conclusion:**

Fourteen epitopes were predicted as promising vaccine candidates against PPRV from four immunogenic proteins. These epitopes should be validated experimentally through in vitro and in vivo studies.

## 1. Introduction

Small ruminant morbillivirus (previously called peste des petits ruminants virus (PPRV)) is one of the most damaging ruminant diseases. It is among the priority diseases indicated in the FAO-OIE Global Framework for the Progressive Control of Transboundary Animal Diseases (GF-TADs) in the 5-year Action Plan [1, 2]. PPRV is one of the top ten diseases in sheep and goats that are having a high impact on the poor rural small ruminant farmers [3]. The disease is considered an acute and highly contagious viral disease with a high morbidity and mortality rate in small ruminants, such as goats and sheep and related wild animals [4, 5]. The disease is characterized by high fever, depression, anorexia, ocular and nasal discharge, pneumonia, necrosis and ulceration of mucous membranes, and inflammation of the gastrointestinal tract leading to severe diarrhea [6, 7]. It causes high death rates in goats and sheep up to 100% and 90%, respectively. However, sheep can be subclinically infected and play a major role in the silent spread of PPRV over large distances and across borders [1]. The disease is widely distributed in Africa, on the Arabian Peninsula, and in the Middle East and Asia [5, 8, 9]. Morbilliviruses are rapidly inactivated at environmental temperature by solar radiation and desiccation. This indicated that the transmission occurred by direct contact with infected animals or their excretions. Transmission of PPRV occurs primarily by droplet infection but may also occur by ingestion of contaminated feed or water [6].

PPRV is an enveloped single strand of negative sense RNA virus, belonging to the genus Morbillivirus, in the family Paramyxoviridae which is closely related to *rinderpest virus* (RPV), *canine distemper virus* (CDV), and *measles virus* (MeV) [5, 10, 11]. The genome of morbilliviruses is organized into six transcriptional units encoding six structural proteins. These structural proteins include the nucleoprotein (N protein), matrix protein (M protein), polymerase or large protein (L protein), phosphoprotein (P protein), and two envelope glycoproteins, the haemagglutinin protein (H protein) and the fusion protein (F protein) [12–14]. The N protein played an important role in the viral life cycle, interacting with both viral and cellular proteins. It also interacted with the viral RNA to form the nucleocapsid structures seen in both the virions and infected cells [13]. The viral L and P proteins interact with the nucleocapsids to form the functional transcription/replication unit of the virion [13]. The C-termini of morbillivirus N proteins also interacted with cellular regulatory proteins such as heat shock protein Hsp72, interferon regulator factor- (IRF-) 3, and a novel cell surface receptor (genetically engineered receptor) [13]. The F protein facilitated the virus penetration of the host cell membrane. This protein is also critical for the induction of an effective protective immune response [15]. The M protein of paramyxoviruses forms an inner coat to the viral envelope and thus serves as a bridge between the surface viral glycoproteins and the ribonucleoprotein core. By virtue of its position, M appeared to play a central role in viral assembly by formation of new virions which were liberated from the infected cell by budding [16, 17]. Interaction of the PPRV H and F proteins with the host plasma membrane led to viral entry by binding of the H protein to receptors [17]. Generally, the protective cell-mediated and humoral immune responses against morbilliviruses are directed mainly against H, F, M, and N proteins. Moreover, PPRV is genetically grouped into four distinct lineages (I, II, III, and IV) based on the analysis of the fusion (F) gene. This classification of PPRV into lineages has broadened the understanding of the molecular epidemiology and worldwide movement of PPR viruses [7, 18–20].

Vaccination is the main tool for controlling and eradicating the PPR virus [12]. Despite the fact that live attenuated vaccines have been widely used to protect small ruminants against circulating PPRV [1, 3, 7], the continuous spread of PPR disease indicated two possible hypotheses. The first is the emergence of new PPRV strains with new genetic makeup and greater fitness in the face of vaccine-elicited protection. The second is the lapses in regulatory control that ultimately lead to movement of diseased/infected individuals across the region/state/country without proper monitoring and surveillance [1].

The advances made in the field of immunoinformatics tools coinciding with the knowledge on the host immune response lead to new disciplines in vaccine design against diseases via computer in silico epitope predictions. The epitope-driven vaccine is a new concept that is being successfully applied in multiple studies, particularly to the development of vaccines targeting conserved epitopes in variable or rapidly mutating pathogens [21–23]. The identification of specific epitopes derived from infectious disease has significantly advanced the development of peptide-based vaccines. Peptides elicited more desirable manipulation of immune response through the use of the B cell epitopes. These epitopes mainly induce antibody production from B cells and cellular response and cytokine secretion from T cells. The approach regarding the molecular basis of antigen recognition and HLA binding motifs to host class I and class II MHC proteins is highly supported by the immunoinformatics which aids in designing epitope-based vaccine motifs that serve as therapeutic candidates for many infectious diseases [24].

The main objective of this study was to analyze multiple immunogenic proteins from the PPR genome for designing a safe multiepitope vaccine using immunoinformatics tools present in the Immune Epitope Database (IEDB). These proteins include haemagglutinin protein (H), matrix protein (M), fusion protein (F), and nucleoprotein (N) sequences of PPRV strains reported in the (NCBI) database.

## 2. Materials and Methods

### 2.1. Sequence Retrieval

Four immunogenic protein sequences of PPRV (updated August 2018) were retrieved from GenBank of the National Center for Biotechnology Information (NCBI) (http://www.ncbi.nlm.nih.gov/protein) in Oct. 2018. These included 82 sequences from the haemagglutinin protein (H protein), 67 sequences from the matrix protein (M protein), 94 sequences from the fusion protein (F protein), and 80 sequences from the nucleoprotein (N protein). All sequences were retrieved in FASTA format. The retrieved sequences, their accession numbers, and geographical locations are listed in Tables 1–4.

### 2.2. Phylogenetic Evolution

A phylogenetic tree of the retrieved sequences of each immunogenic protein was constricted using MEGA7.0.26 (7170509) software [25]. Each protein tree was constructed using the maximum likelihood parameter in the software.

### 2.3. Multiple Sequence Alignment

The complete protein sequences of each immunogenic protein of PPRV were aligned via BioEdit software (version 7.2.5) to generate a multiple sequence alignment (MSA) with the ClustalW tool [26].

### 2.4. Epitope Prediction

Several immunobioinformatics tools were used for prediction of multiple epitopes from the four immunogenic proteins of PPRV. Tools from the Immune Epitope Database analysis resource (http://www.iedb.org/) [27] were used to analyze the immunogenic proteins. The input was the reference sequences of H protein (YP_133827.2), M protein (YP_133825.1), F protein (YP_133826.1), and N protein (YP_133821.1). They were submitted to Epitope Analysis Resources to predict B and T cell epitopes. The predicted epitopes were further investigated in aligned retrieved sequences for conservancy to identify the proposed candidate epitopes.

#### 2.4.1. B Cell Epitope Prediction

Epitopes that interacted with the B lymphocytes are a discrete part from the antigenic molecule that is recognized by the B cell receptor and elicited immunoglobulin production. These predicted epitopes are characterized by their surface accessibility and their antigenic reactivity with the immunoglobulins of the humoral immunity [24]. Epitope prediction tools of the Immune Epitope Database (IEDB) at http://tools.iedb.org/bcell/ [27] were used for this purpose. Linear B cell epitopes were predicted by BepiPred linear epitope prediction (http://tools.iedb.org/bcell/result/) [28]. The Emini surface accessibility prediction tool was performed to detect the surface accessible epitopes (http://tools.iedb.org/bcell/) [29], while prediction of antigenic epitopes was performed to identify the antigenic determinants on proteins based on the physicochemical properties of amino acid residues using the Kolaskar and Tongaonkar antigenicity method (http://tools.immuneepitope.org/bcell/) [30].

#### 2.4.2. Cytotoxic T Lymphocyte Epitope Prediction

IEDB tools (http://tools.iedb.org/mhci/) were used to predict different cytotoxic T cell (CTL) epitopes that bind to the major histocompatibility complex class I alleles (MHC class I) [31]. Analysis was done using cow alleles (BoLA-D18.4, BoLA-HD6, BoLA-JSP.1, BoLA-T2a, BoLA-T2b, and BoLA-T2c). An artificial neural network (ANN) was used to predict the binding affinity [32, 33]. The peptide length for all selected epitopes was set to 9 amino acids (9mers). Percentile rank required for the peptide's binding to the specific MHC-I molecules was set in the range from 1 to 3.

### 2.5. Homology Modeling

#### 2.5.1. The Three-Dimensional (3D) Structures of the Reference Sequences of PPRV

The prediction of the three-dimensional (3D) structure of H, M, and F protein reference sequences of PPRV was performed using the RaptorX structure prediction server (http://raptorx.uchicago.edu/StructurePrediction/predict/) [34–36], while the N protein sequence was submitted to the SPARKS-X server (http://sparks-lab.org/yueyang/server/SPARKS-X/) [37]. The 3D structure of each protein reference sequence was later treated with Chimera software 1.8 to show the position of proposed epitopes [38].

## 3. Results and Discussion

The validity and benefits of peptide vaccines designed by bioinformatics tools had been verified by appreciable research [24]. The availability of the complete genome, proteome sequences, and pathogenesis of many pathogenic microorganisms contributed to the production of a vaccine through bioinformatics [24, 39]. In this study, the predicted epitopes from B and T lymphocytes would help in the development of a more effective, reliable, preventive, and therapeutic vaccine against the PPRV than the conventional methods.

### 3.1. Phylogenetic Evolution

**Figure 1:**
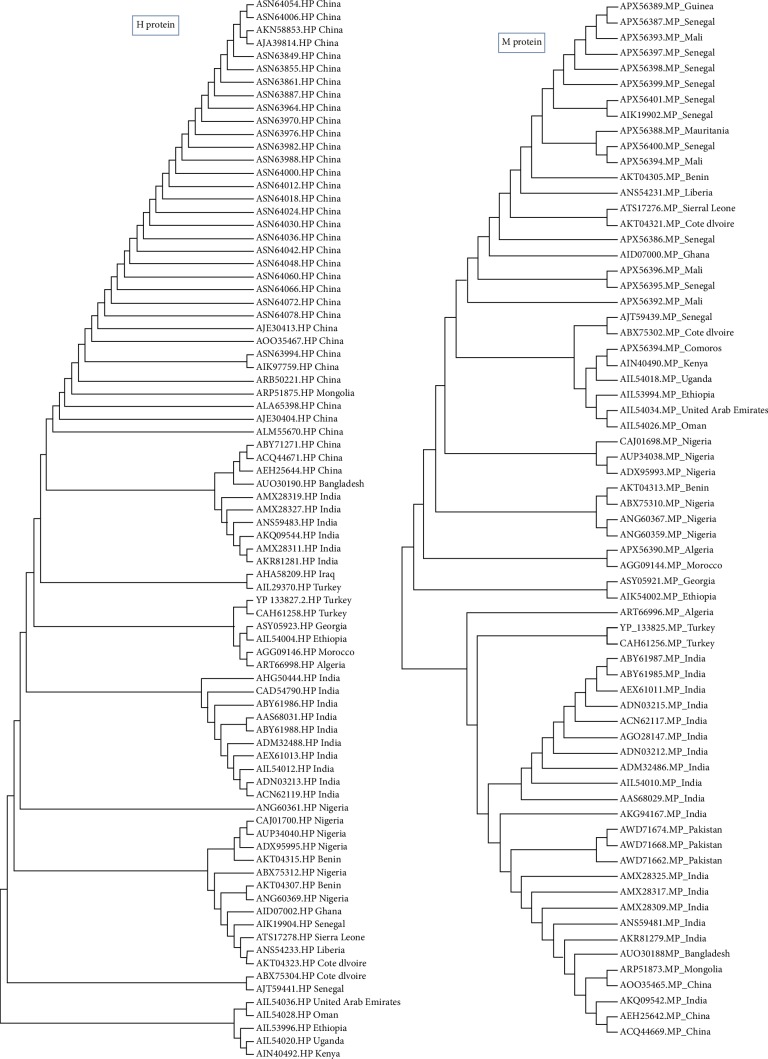
Phylogenetic tree of retrieved strains of H and M proteins. The retrieved strains demonstrated divergence in their common ancestors.

**Figure 2:**
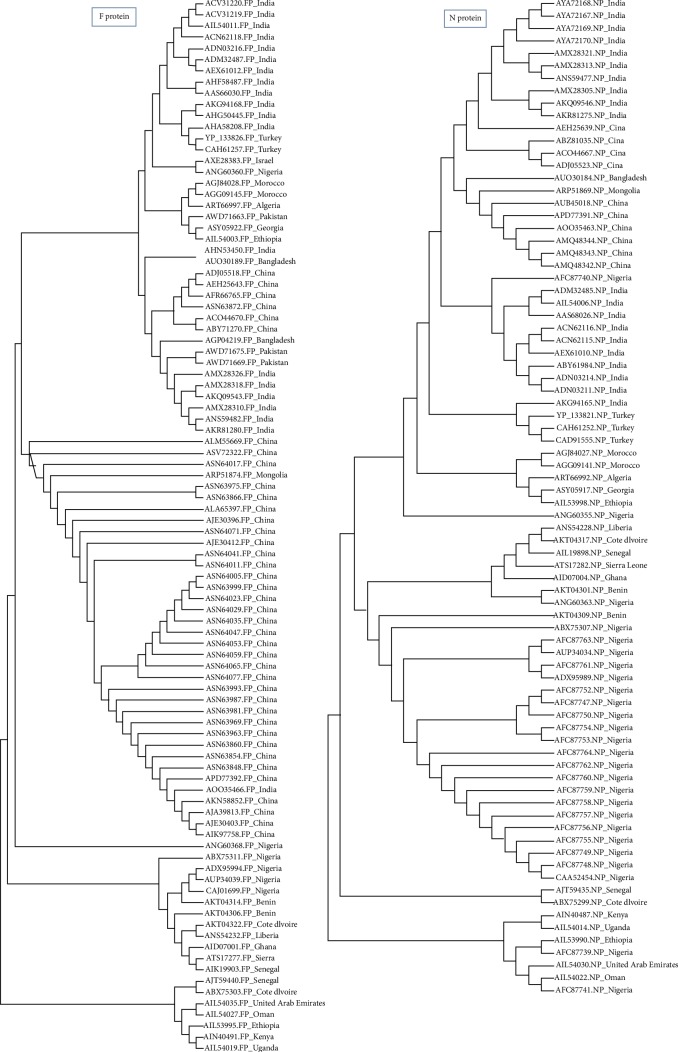
Phylogenetic tree of retrieved strains of F and N proteins. The retrieved strains demonstrated divergence in their common ancestors.

A phylogenetic tree was constructed using MEGA7.0.26 (7170509). The evolutionary divergence among each protein was analyzed. As shown in Figure 1, the retrieved strains of the H protein revealed that Asian strains were clustered together as well as the European and African strains. However, strains from the United Arab Emirates and Oman were closely related to African strains (namely to Ethiopian strains). With regard to the phylogeny of the M protein strains, the African strains were also clustered together, but among them, the Oman and United Arab Emirates strains were observed to be close to the Ethiopian strains same as those of the H protein. This result may indicate the transfer of the H and M strain segments between these countries. Also, some European and Turkish strains were clustered together. As shown in Figure 2, the retrieved strains of F and N proteins from the Asian strains were clustered together with molecular divergence among them as well as the strains retrieved from the African countries. Also, the Omanis and Emiratis strains showed close relationship to the African strains. These results indicated that these strain segments were widely distributed in Africa, Asia, Europe, and the Arab region.

### 3.2. Sequence Alignment

**Figure 3:**
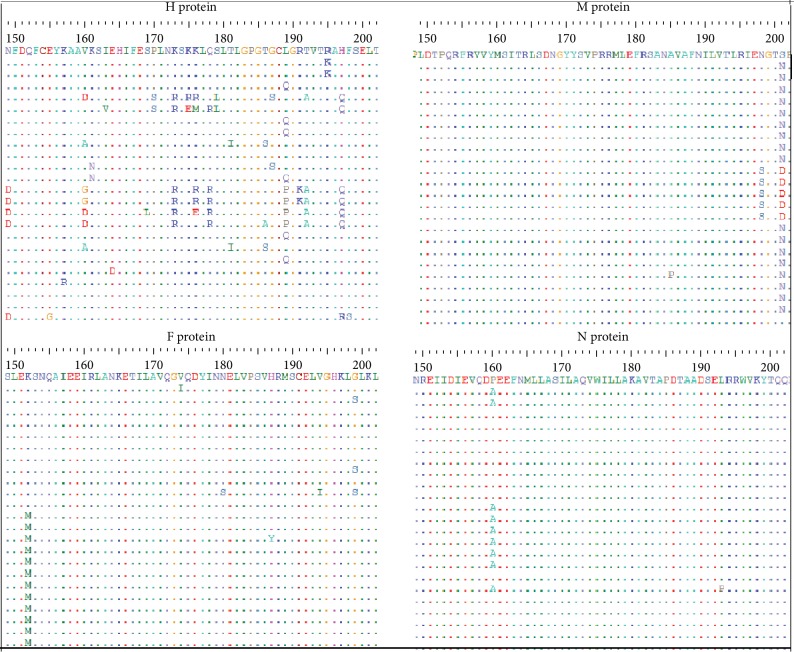
Multiple sequence alignment (MSA) of the retrieved strains of H, M, F, and N proteins using BioEdit software and ClustalW. Dots indicate the conservancy of the retrieved strains, and letters within the aligned sequences indicate no conservancy (mutation) in the amino acid.

Multiple sequence alignment was performed using ClustalW in BioEdit software. As shown in Figure 3, the aligned sequences of each of the four analyzed proteins (H, M, F, and N proteins) showed considerable conservancy among the retrieved strains. However, some regions exhibited differences (mutations) in some amino acids in various sequences.

### 3.3. Prediction of B Cell Epitopes

B cell epitope prediction methods aimed are at identifying the antigens recognized by B lymphocytes to initiate humoral immunity [24]. The important criteria for selecting a potential epitope for vaccine development are surface accessibility, hydrophobicity, flexibility, and antigenicity [40]. The predicted epitopes should be located on the surface of the cells so that it is more accessible for both the humoral and the cellular immune systems. Antigenicity also is one of the important features of an antigen for vaccine development [40]. Depending on binding affinity to B lymphocytes, the BepiPred linear epitope prediction method predicted nine linear epitopes from the H protein, eight epitopes from M proteins, and ten epitopes for each of the F and N proteins. Analysis of these linear epitopes for surface accessibility proposed seven surface epitopes from each of the H and F proteins, six epitopes from the M protein, and four epitopes from the N protein.

**Figure 4:**
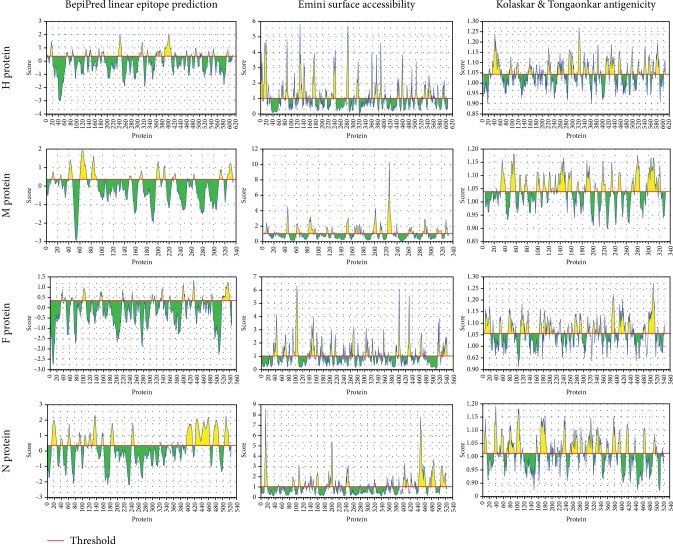
Prediction of B cell epitopes by different IEDB scales (BepiPred linear epitope prediction, Emini surface accessibility, and Kolaskar and Tongaonkar antigenicity prediction) for H, M, F, and N proteins. Regions above the threshold (red line) were proposed as a part of the B cell epitope while regions below the threshold (red line) were not.

As shown in Figure 4, the threshold values were 0.350 and 1.000 for all epitopes predicted through the BepiPred linear epitope (conserved epitopes) and Emini prediction methods (surface accessibility), respectively. The antigenicity prediction method proposed only two epitopes for all test immunogenic proteins of PPRV. Also, Figure 4 shows that the antigenic epitopes were predicted from H, M, F, and N proteins using the Kolaskar and Tongaonkar antigenicity method under threshold values of 1.014, 1.037, 1.054, and 1.014, respectively. However, no epitopes successfully passed the threshold for the F and N proteins.

**Figure 5:**
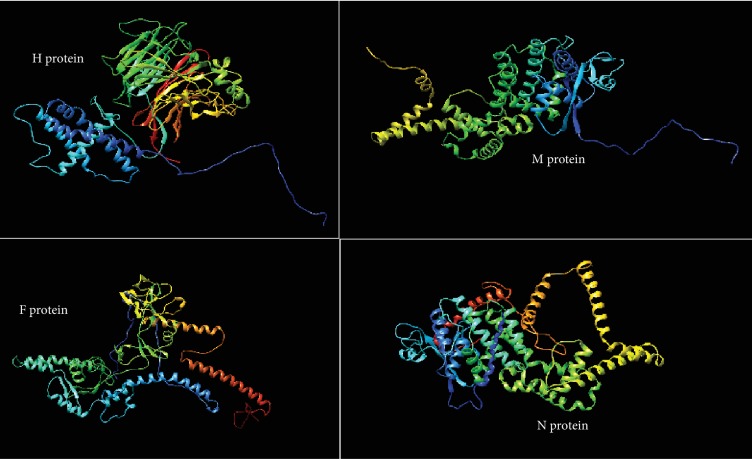
The prediction of the three-dimensional (3D) structure of H, M, and F protein reference sequences of PPRV was performed using the RaptorX structure prediction server, while the N protein sequence was submitted to the SPARKS-X server.

**Figure 6:**
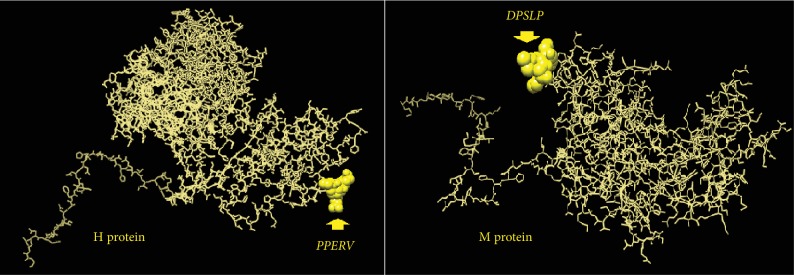
The positions of the proposed B cell epitopes in the 3D structure of the reference sequences of PPRV H and M proteins.

Only one epitope from each of the H and M proteins successfully overlapped all the B cell antigenic index prediction methods. Namely, these epitopes were *_142_PPERV_146_* from the H protein and *_63_DPLSP_67_* from the M protein. The 3D structure of the four proteins (H, M, F, and N) is shown in Figure 5. The positions of the best B cells that predicted epitopes from the H and M proteins are demonstrated in Figure 6. The overall predicted epitopes from the four proteins are illustrated in Table 5.

### 3.4. Prediction of CTL Epitopes That Interacted with MHC Class I (BoLA Alleles)

CD8+ and CD4+ T cells have a principal role in the stimulation of immune response as well as antigen-mediated clonal expression of the B cell [14]. Unfortunately, the bovine genome project did not assemble a complete sequence of the bovine MHC-II locus [41–43]. Thus, the analysis was completed with BoLA MHC-I alleles only. Cell-mediated immunity induced by cytotoxic T lymphocytes (CTLs) is vital for the defense against viral diseases. CTLs are responsible for the immune elimination of intracellular pathogens such as viruses because these cells recognize the presented endogenous antigenic peptides by the MHC class I molecules [44].

In this study, MHC-I binding prediction methods using the IEDB database predicted different CTL epitopes that strongly interacted with various BoLA alleles. The fusion (F) protein proposed a higher number of predicted epitopes with strong interaction with BoLA alleles. Ten epitopes were proposed based on the number of the interacted alleles. The best one was *_358_STKSCARTL_366_* that associated with five alleles, followed by *_442_IDLGPAISL_450_* and *_340_SQNALYPMS_348_* as they linked to three alleles each. However, seven epitopes, namely, *_339_CSQNALYPMS_347_*, *_336_GTVCSQNAL_344_*, *_279_IAYPTLSEI_287_*, *_230_LSYALGGDI_238_*, *_136_SLMNSQAIE_144_*, *_283_TLSEIKGVI_291_*, and *_314_YVATQGYLI_322_* were predicted to interact with two alleles.

The nucleoprotein (N) also displayed strong interaction activity with BoLA alleles. Seven epitopes were proposed with strong interaction with BoLA alleles. The top N protein epitope was *_490_RSAEALFRL_498_* which was associated with five alleles, followed by two epitopes, namely, _2_*ATLLKSLAL*_10_, and *_304_QQLGEVAPY_312_* that linked to four alleles each. In addition to that, four epitopes *_3_TLLKSLALF_11_*, *_356_YFDPAYFRL_364_*, *_360_AYFRLGQEM_368_*, and *_412_PRQAQVSFL_420_* interacted with three bovine alleles each. Surprisingly, these two proteins (F and N) achieved promising results in CTL prediction methods, although they failed to predict any epitope carrying all the ideal traits in B cells.

**Figure 7:**
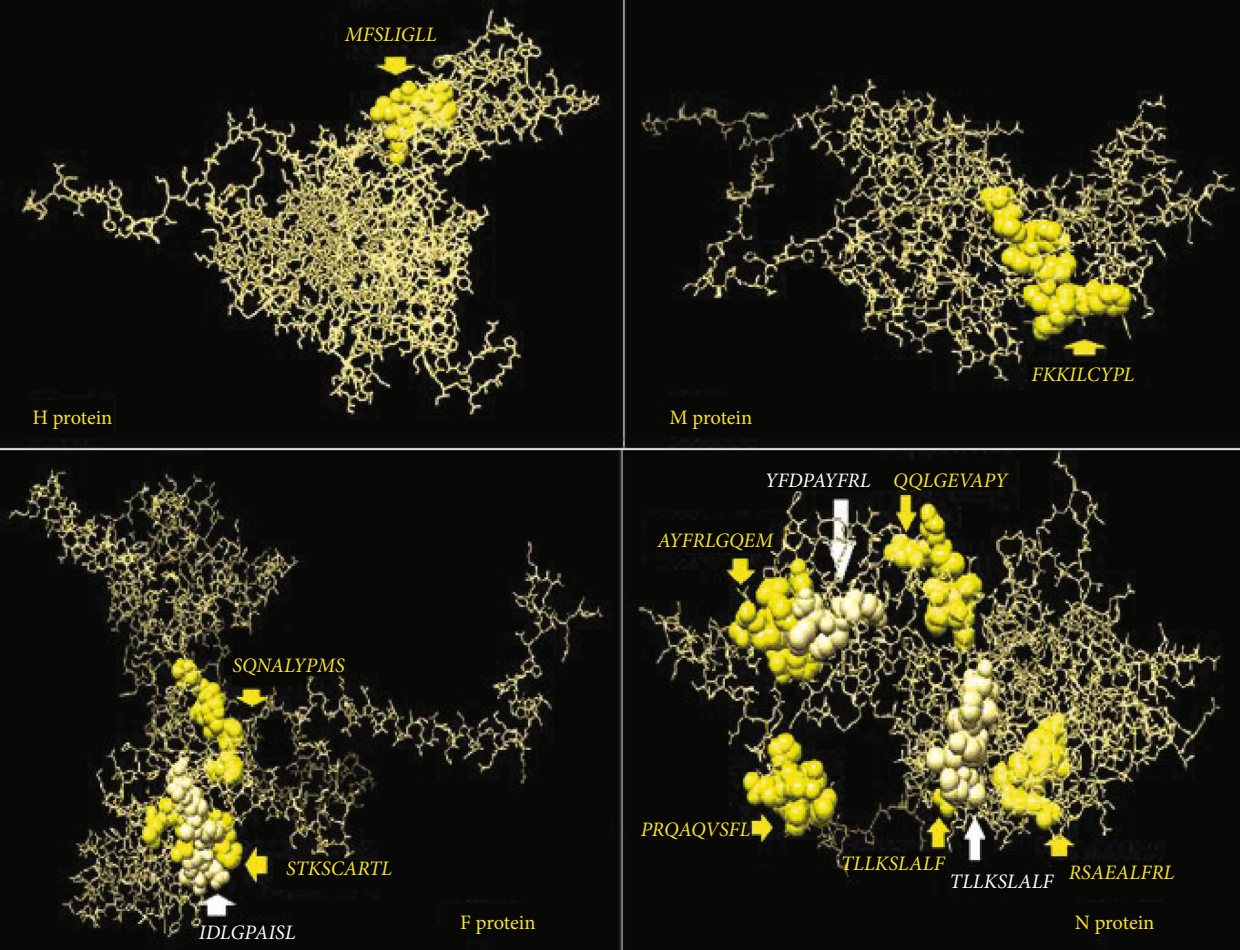
The positions of the predicted T cell epitopes in the 3D structure of the reference sequences of PPRV H, M, F, and N proteins.

The haemagglutinin (H) protein predicted five CTL epitopes, but one epitope was predicted as the best peptide, *_45_MFLSLIGLL_53_*, as it linked to four BoLA alleles, followed by four peptides that interacted with two alleles each. They were *_113_DLVKFISDK_121_*, *_405_GRIPAYGVI_413_*, *_52_LLAIAGIRL_60_*, and *_44_VMFLSLIGL_52_*. However, this protein showed a somewhat satisfactory result in B and T cell prediction methods. The M protein showed unsatisfactory results in CTL prediction methods different from that predicted by B cell methods. The results suggested only one epitope; *_276_FKKILCYPL_284_* interacted with only two alleles. The overall epitopes that were proposed to interact with CTL alleles are illustrated in Table 6 for all proteins. The positions of the best CTL-predicted epitopes in their immunogenic protein structure are shown in Figure 7.

Vaccination is considered the most effective way of controlling PPR. The infection by morbillivirus is associated with severe immunosuppression that is characterized by a massive virus-specific immune response. Protection is mediated by cell-mediated and humoral immune responses directed mainly against particular proteins in the viral structure. These proteins included H, F, and N proteins [45–47]. It was reported that the envelope glycoproteins H and F of PPRV demonstrated a protective and neutralizing antibody response [3, 48–50]. In this study, using the immunoinformatics prediction methods, the H protein demonstrated affinity to interact with B cells that was characterized by antibody production. This result coincided with the previously published reports [3, 48–50], while the F protein failed to interact with B cells; i.e., no epitopes from the F protein had passed the threshold of the B cell prediction methods. However, this protein revealed multiple predicted epitopes that demonstrated high affinity to the alleles of CTLs. The M protein which is believed to play a very significant role in morbillivirus assembly and budding by concentrating the F, H, and N proteins at the virus-assembly site [16, 17] showed moderate affinity to B cells. One epitope from the M protein as well as the H protein was predicted as a B cell epitope. Moreover, the M protein revealed multiple epitopes that interacted with CTLs of the cell-mediated immunity. This result indicated that the M protein besides its role in the virus assembly may also contain antigenic determinants that could be elected as vaccine candidates.

In addition to that, cell-mediated immunity plays a role in protection against the viral infection. Despite the N protein being the most frequent viral protein in PPRV, it does not induce a neutralizing antibody response in the host [50]. However, it has been found to induce a strong cell-mediated immune response, which is believed to contribute to protection. Here, in this report, the same result was obtained. The N protein demonstrated no affinity to elicit the humoral immune response. However, it showed favorable affinity to interact with a cell-mediated response. It is noteworthy that five out of seven epitopes predicted from the nucleoprotein of PPRV in this study were found to be proposed by another *in silico* study using mouse alleles and NetMHCI methods [51]. The proposed epitopes from that study were *ATLLKSLAL*, *TLLKSLALF*, *YFDPAYFRL*, *AYFRLGQEM*, and *RSAEALFRL*. Thus, the predictions for the different epitopes that bound to different alleles particularly from the N protein of PPRV were somewhat in agreement regardless of the alleles (cow and mouse alleles) and algorithm used (ANN, NetMHCI).

In general, epitope-based vaccines that are chemically well-characterized have become desirable candidate vaccines due to their relative ease of production and construction, chemical stability, and lack of infectious potential [52]. Many *in silico* studies have shown the value of using prediction programs to evaluate the efficiency of binding of putative epitopes to various human and animal alleles [33, 52–55].

## 4. Conclusion

This study focused mainly on the production of a peptide vaccine against H, M, F, and N proteins of PPRV using immunoinformatics tools. Epitopes that showed conservancy and high binding affinities to many MHC alleles are considered the best candidates for *in vitro* and *in vivo* testing. Epitopes that were predicted from B cell prediction methods like *_142_PPERV_146_* and *_305_TVTL_308_* from the H protein and *_63_DPLSP_67_* and *_64_PLSP_67_* from the M protein could act as good B cell epitopes to induce humoral immunity. While the F and N proteins failed to fulfill all B cell indexes used in this study for the prediction of promising epitopes, however, these proteins predicted epitopes that interacted with various BoLA MHC-I alleles. For instance, the best epitopes were predicted from F (*_358_STKSCARTL_366_*) and N (*_490_RSAEALFRL_498_*) proteins as they interacted with five MHC-I BoLA alleles, followed by *_45_MFLSLIGLL_53_* proposed from the H protein and linked with four alleles, while the *_276_FKKILCYPL_284_* epitope was predicted from the M protein linked with only two alleles. Although bioinformatics studies have been established to facilitate the peptide design, not all peptides that are predicted *in silico* are optimally immunogenic *in vivo* and it remains necessary to test the expected peptides *in vivo* to ensure that the T cell responses are elicited.

## Figures and Tables

**Table 1:** Retrieved strains of the H protein of PPRV with their date of collection, accession numbers, and geographical regions.

No.	Accession number	Country	Year	No.	Accession number	Country	Year	No.	Accession number	Country	Year
1	AEH25644	China	2011	29	ATS17278	Sierra Leone	2017	57	ASN64042	China	2017
2	ABY71271	China	2008	30	AMX28327	India	2017	58	ASN64036	China	2017
3	AAS68031	India	2009	31	AMX28319	India	2017	59	ASN64030	China	2017
4	ABX75304	Cote d'Ivoire	2008	32	AMX28311	India	2017	60	ASN64024	China	2017
5	ABX75312	Nigeria	2008	33	ANS54233	Liberia	2016	61	ASN64018	China	2017
6	ADM32488	India	2012	34	AKT04315	Benin	2016	62	ASN64012	China	2017
7	AEX61013	India	2012	35	AKT04307	Benin	2016	63	ASN64006	China	2017
8	ASY05923	Georgia	2017	36	AKR81281	India	2015	64	ASN64000	China	2017
9	ARB50221	China	2017	37	AKT04323	Cote d'Ivoire	2015	65	ASN63994	China	2017
10	ANS59483	India	2016	38	AJT59441	Senegal	2015	66	ASN63988	China	2017
11	AKQ09544	India	2015	39	AID07002	Ghana	2015	67	ASN63982	China	2017
12	AIL54036	UAE	2014	40	AIN40492	Kenya	2014	68	ASN63976	China	2017
13	AIL54028	Oman	2014	41	AHG50444	India	2014	69	ASN63970	China	2017
14	AIL54020	Uganda	2014	42	ACQ44671	China	2011	70	ASN63964	China	2017
15	AIL53996	Ethiopia	2014	43	ABY61988	India	2008	71	ASN63867	China	2017
16	AIL54012	India	2014	44	ABY61986	India	2008	72	ASN63861	China	2017
17	AIL54004	Ethiopia	2014	45	CAH61258	Turkey	2005	73	ASN63855	China	2017
18	CAD54790	India	2004	46	ANG60369	Nigeria	2016	74	ASN63849	China	2017
19	AHA58209	Iraq	2018	47	ANG60361	Nigeria	2016	75	ART66998	Algeria	2017
20	ARP51875	Mongolia	2018	48	AJE30413	China	2015	76	ALM55670	China	2016
21	AOO35467	China	2018	49	AJE30404	China	2015	77	ALA65398	China	2015
22	AIL29370	Turkey	2014	50	AUP34040	Nigeria	2018	78	AKN58853	China	2015
23	AGG09146	Morocco	2014	51	ASN64078	China	2015	79	AJA39814	China	2015
24	ADN03213	India	2013	52	ASN64072	China	2017	80	AIK97759	China	2014
25	ACN62119	India	2015	53	ASN64066	China	2017	81	AIK19904	Senegal	2014
26	CAJ01700	Nigeria	2005	54	ASN64060	China	2017	82	ADX95995	Nigeria	2011
27	YP_133827	Turkey	2018	55	ASN64054	China	2017				
28	AUO30190	Bangladesh	2018	56	ASN64048	China	2017				

**Table 2:** Retrieved strains of the matrix protein of PPRV with their date of collection, accession numbers, and geographical regions.

No.	Accession number	Country	Year	No.	Accession number	Country	Year	No.	Accession number	Country	Year
1	YP_133825	Turkey	2018	24	APX56396	Mali	2013	47	AIL54018	Uganda	2014
2	ANS54231	Liberia	2016	25	APX56395	Senegal	2015	48	AIL54010	India	2014
3	AKT04313	Benin	2016	26	APX56394	Comoros	2005	49	AIL53994	Ethiopia	2014
4	AKT04305	Benin	2016	27	APX56393	Mali	2018	50	AIL54002	Ethiopia	2014
5	AMX28325	India	2017	28	APX56392	Mali	2018	51	AGO28147	India	2013
6	AMX28317	India	2017	29	APX56391	Mali	2018	47	AIL54018	Uganda	2014
7	ANS59481	India	2016	30	APX56390	Algeria	2018	52	AGG09144	Morocco	2013
8	AKR81279	India	2015	31	APX56389	Guinea	2018	53	AEH25642	China	2011
9	CAJ01698	Nigeria	2005	32	APX56388	Mauritania	2018	54	ACQ44669	China	2011
10	ADN03215	India	2015	33	APX56387	Senegal	2018	55	ABY61987	India	2008
11	ADN03212	India	2012	34	APX56386	Senegal	2018	56	ABY61985	India	2008
12	ADM32486	India	2012	35	AUO30188	Bangladesh	2018	57	CAH61256	Turkey	2005
13	ACN62117	India	2012	36	ATS17276	Sierra Leone	2017	58	AOO35465	China	2016
14	AEX61011	India	2012	37	ASY05921	Georgia	2017	59	ABX75310	Nigeria	2008
15	AWD71674	Pakistan	2018	38	ARP51873	Mongolia	2017	60	ABX75302	Cote d'Ivoire	2008
16	AWD71668	Pakistan	2018	39	AKQ09542	India	2015	61	AUP34038	Nigeria	2018
17	AWD71662	Pakistan	2018	40	AKT04321	Cote d'Ivoire	2015	62	ART66996	Algeria	2017
18	AMX28309	India	2017	41	AJT59439	Senegal	2015	63	AIK19902	Senegal	2014
19	APX56401	Senegal	2018	42	AKG94167	India	2015	64	ADX95993	Nigeria	2011
20	APX56400	Senegal	2018	43	AID07000	Ghana	2015	65	AAS68029	India	2009
21	APX56399	Senegal	2018	44	AIN40490	Kenya	2014	66	ANG60367	Nigeria	2016
22	APX56398	Senegal	2014	45	AIL54034	UAE	2014	67	ANG60359	Nigeria	2016
23	APX56397	Senegal	2014	46	AIL54026	Oman	2014				

**Table 3:** Retrieved strains of the fusion protein of PPRV with their date of collection, accession numbers, and geographical region.

No.	Accession number	Country	Year	No.	Accession number	Country	Year	No.	Accession number	Country	Year
1	YP_133821	Turkey	2018	28	AMQ48343	China	2016	55	AFC87747	Nigeria	2012
2	AIN40487	Kenya	2014	29	AMQ48342	China	2016	56	AFC87741	Nigeria	2012
3	AYA72170	India	2018	30	AKQ09546	India	2015	57	AFC87740	Nigeria	2012
4	AYA72169	India	2018	31	AKG94165	India	2015	58	AFC87739	Nigeria	2012
5	AYA72168	India	2018	32	AIL54030	UAE	2014	59	ABZ81035	China	2008
6	AYA72167	India	2018	33	AIL54022	Oman	2014	60	AEX61010	India	2012
7	ACN62116	India	2012	34	AIL54014	Uganda	2014	61	AEH25639	China	2011
8	ACN62115	India	2012	35	AIL53990	Ethiopia	2014	62	ACQ44667	China	2011
9	AUO30184	Bangladesh	2018	36	AIL54006	India	2014	63	CAH61252	Turkey	2005
10	AUB45018	China	2017	37	AIL53998	Ethiopia	2014	64	CAD91555	Turkey	2003
11	ATS17282	Sierra Leone	2017	38	AGJ84027	Morocco	2013	65	CAA52454	Nigeria	2005
12	ANS54228	Liberia	2016	39	AFC87764	Nigeria	2012	66	AMX28321	India	2017
13	AKT04301	Benin	2016	40	AFC87763	Nigeria	2012	67	AMX28313	India	2017
14	AKT04317	Cote d'Ivoire	2015	41	AFC87762	Nigeria	2012	68	AMX28305	India	2017
15	AJT59435	Senegal	2015	42	AFC87761	Nigeria	2012	69	ANS59477	India	2016
16	ABX75299	Cote d'Ivoire	2008	43	AFC87760	Nigeria	2012	70	AKT04309	Benin	2016
17	ABY61984	India	2008	44	AFC87759	Nigeria	2012	71	AKR81275	India	2015
18	ABX75307	Nigeria	2008	45	AFC87758	Nigeria	2012	72	AGG09141	Morocco	2013
19	ART66992	Algeria	2017	46	AFC87757	Nigeria	2012	73	ADJ05523	China	2010
20	APD77391	China	2016	47	AFC87756	Nigeria	2012	74	AAS68026	India	2009
21	ADN03214	India	2012	48	AFC87755	Nigeria	2012	75	ANG60363	Nigeria	2016
22	ADN03211	India	2012	49	AFC87754	Nigeria	2012	76	ANG60355	Nigeria	2016
23	ADM32485	India	2012	50	AFC87753	Nigeria	2012	77	AUP34034	Nigeria	2018
24	ASY05917	Georgia	2017	51	AFC87752	Nigeria	2012	78	AIK19898	Senegal	2014
25	ARP51869	India	2017	52	AFC87750	Nigeria	2012	79	ADX95989	Nigeria	2011
26	AOO35463	China	2017	53	AFC87749	Nigeria	2012	80	AID07004	Ghana	2015
27	AMQ48344	China	2016	54	AFC87748	Nigeria	2012				

**Table 4:** Retrieved strains of the nucleoprotein of PPRV with their date of collection, accession numbers, and geographical regions.

No.	Accession number	Country	Year	No.	Accession number	Country	Year	No.	Accession number	Country	Year
1	AHA58208	Iraq	2018	33	ASN63969	China	2017	65	AIL54019	Uganda	2014
2	AOO35466	China	2016	34	ASN63963	China	2017	66	AIL53995	Ethiopia	2014
3	AGJ84028	Morocco	2013	35	ASN63872	China	2017	67	AIK97758	China	2014
4	CAJ01699	Nigeria	2005	36	ASN63866	China	2017	68	AIK19903	Senegal	2014
5	AHN53450	India	2014	37	ASN63860	China	2017	69	AIL54011	India	2014
6	ADN03216	India	2012	38	ASN63854	China	2017	70	AIL54003	Ethiopia	2014
7	ADM32487	India	2012	39	ASN63848	China	2017	71	AHG50445	India	2014
8	ACN62118	India	2012	40	ARP51874	Mongolia	2017	72	AGP04219	Bangladesh	2013
9	AEX61012	India	2012	41	AMX28326	India	2017	73	AGG09145	Morocco	2013
10	ADX95994	Nigeria	2011	42	AMX28318	India	2017	74	AFR66765	China	2012
11	YP_133826	Turkey	2017	43	AMX28310	India	2017	75	ACV31220	India	2012
12	ATS17277	Sierra	2017	44	APD77392	China	2016	76	ACV31219	India	2012
13	ASY05922	Georgia	2017	45	ANS59482	India	2016	77	ACQ44670	China	2011
14	ASV72322	China	2017	46	AHF58487	India	2016	78	ADJ05518	China	2010
15	ASN64077	China	2017	47	ALM55669	China	2016	79	CAH61257	Turkey	2005
16	ASN64071	China	2017	48	AKT04314	Benin	2016	80	AXE28383	Israel	2018
17	ASN64065	China	2017	49	AKT04306	Benin	2016	81	AWD71675	Pakistan	2018
18	ASN64059	China	2017	50	ALA65397	China	2015	82	AWD71669	Pakistan	2018
19	ASN64053	China	2017	51	AKN58852	China	2015	83	AWD71663	Pakistan	2018
20	ASN64047	China	2017	52	AKR81280	India	2015	84	AUP34039	Nigeria	2018
21	ASN64041	China	2017	53	AKQ09543	India	2015	85	AUO30189	Bangladesh	2018
22	ASN64035	China	2017	54	AKT04322	Cote d'Ivoire	2015	86	ANG60368	Nigeria	2016
23	ASN64029	China	2017	55	AJT59440	Senegal	2015	87	ANG60360	Nigeria	2016
24	ASN64023	China	2017	56	AKG94168	India	2015	88	ANS54232	Liberia	2016
25	ASN64017	China	2017	57	AJA39813	China	2015	89	ART66997	Algeria	2017
26	ASN64011	China	2017	58	AID07001	Ghana	2015	90	ABX75303	Cote d'Ivoire	2008
27	ASN64005	China	2017	59	AJE30412	China	2015	91	ABX75311	Nigeria	2008
28	ASN63999	China	2017	60	AJE30403	China	2015	92	ABY71270	China	2008
29	ASN63993	China	2017	61	AJE30396	China	2015	93	AAS68030	India	2009
30	ASN63987	China	2017	62	AIN40491	Kenya	2014	94	AEH25643	China	2011
31	ASN63981	China	2017	63	AIL54035	UAE	2014				
32	ASN63975	China	2017	64	AIL54027	Oman	2014				

**Table 5:** B cell epitope prediction from H, M, F, and N proteins; the position of peptides is according to the position of amino acids in the protein of the PPR virus.

H protein	Peptide	Start	End	Length	Emini 1.000	Kolaskar 1.041
1	PHNK	16	19	4	2.683	0.969
2	SIDHQ	83	87	5	1.169	1.03
3	PPERV^#^	142	146	5	1.904	1.047
4	TVTL	305	308	4	0.505	1.113
5	TLGG	330	333	4	0.462	0.977
6	EANWVVPSTDVRDLQ	362	376	15	1.022	1.039
7	KTRPPSFCNGTG	387	398	12	1.314	0.982
8	GPWSEGRIP	400	408	9	1.023	0.962
9	DVSR	530	533	4	1.29	1.034

M protein	Peptide	Start	End	Length	Emini 1.000	Kolaskar 1.037
1	SAWDV	10	14	5	0.533	1.044
2	GDRK	43	46	4	2.478	0.886
3	EDNDPLSP^∗^	60	67	8	3.167	0.969
4	DPLSP^∗^^#^	63	67	5	1.332	1.051
5	VGRT	69	72	4	0.795	1.01
6	PEEL	87	90	4	1.464	1.004
7	DNGYYS^∗^	167	172	6	2.116	0.975
8	INDD	325	328	4	1.203	0.915

F protein	Peptide	Start	End	Length	Emini 1.000	Kolaskar 1.054
1	TGSA	34	37	4	0.867	0.965
2	SNQA	153	156	4	1.691	0.967
3	SLRDP	216	220	5	2.058	1.013
4	QEWYT	305	309	5	2.625	0.966
5	VFTP	331	334	4	0.643	1.112
6	GTVC	336	339	4	0.255	1.144
7	GSTKS	357	361	5	1.888	0.947
8	QDPDK	402	406	5	5.498	0.948
9	VGSREYPD	428	435	8	2.837	1.01
10	LKPDLTGTSKS	531	541	11	3.269	0.996

N protein	Peptide	Start	End	Length	Emini 1.000	Kolaskar 1.014
1	DKAPTASGSGGAI^∗^	16	28	13	0.249	0.981
2	IPGDSSI	39	45	7	0.345	1.019
3	GDPDINGS	60	67	8	0.735	0.935
4	TDDPDV	92	97	6	1.569	0.992
5	STRSQS	107	112	6	2.397	0.972
6	GADLD^∗^	120	124	5	0.619	0.984
7	VTAPDTAADS	182	191	10	0.594	1.02
8	RTPGNKPR	242	249	8	4.853	0.92
9	KFSA	323	326	4	0.83	1.024
10	RGTGPRQA^∗^	408	415	8	1.69	0.943

^∗^Peptides revealed a higher score if they were shortened in all tools. ^#^Epitopes that passed all the B cell prediction methods and were proposed as B cell epitopes.

**Table 6:** Position of CTL epitopes in the H protein, M protein, F protein, and N protein of PPRV that bind with high affinity with the BoLA class I alleles.

	Peptide	Start	End	Allele	Percentile rank
H protein	DLVKFISDK	113	121	BoLA-T2a	2
				BoLA-T2C	1.9
	FLRVFEIGL	251	259	BoLA-HD6	1
	GRIPAYGVI	405	413	BoLA-D18.4	2.3
				BoLA-T2b	2.1
	LLAIAGIRL	52	60	BoLA-HD6	1.5
				BoLA-T2b	2.3
	LSLIGLLAI	47	55	BoLA-T2a	2.9
	LVKFISDKI	114	122	BoLA-HD6	1.3
	MFLSLIGLL	45	53	BoLA-HD6	2.1
				BoLA-JSP.1	1.7
				BoLA-T2b	1.4
				BoLA-T2C	1.7
	VMFLSLIGL	44	52	BoLA-JSP.1	2.6
				BoLA-T2C	1.7
	WCYHDCLIY	578	586	BoLA-T2a	1.2
	WSEGRIPAY	402	410	BoLA-JSP.1	2.2

M protein	SAWDVKGSI	10	18	BoLA-HD6	1.8
	EELLREATE	88	96	BoLA-T2b	1
	ELLREATEL	89	97	BoLA-HD6	1.3
	PQRFRVVYM	152	160	BoLA-JSP.1	1.7
	HVGNFRRKK	220	228	BoLA-T2a	2.4
	GGIGGTSLH	251	259	BoLA-T2a	2
	LHAQLGFKK	270	278	BoLA-T2a	1.2
	AQLGFKKIL	272	280	BoLA-T2C	2.4
	FKKILCYPL	276	284	BoLA-D18.4	1.2
				BoLA-HD6	1.3
	EFRVYDDVI	316	324	BoLA-HD6	2.6

F protein	AGVALHQSL	129	137	BoLA-T2C	1.7
	ASVLCKCYT	387	395	BoLA-T2a	1.4
	AYPTLSEIK	280	288	BoLA-T2a	2.9
	CSQNALYPM	339	347	BoLA-JSP.1	2.3
				BoLA-T2a	2.5
	DETSCVFTP	326	334	BoLA-T2b	2.7
	DLGPAISLE	443	451	BoLA-T2C	1.3
	EKLDVGTNL	451	459	BoLA-T2b	2.7
	GSTKSCART	357	365	BoLA-T2a	2.6
	GTVCSQNAL	336	344	BoLA-JSP.1	2.5
				BoLA-T2b	1.6
	GVALHQSLM	130	138	BoLA-HD6	2.1
	IAYPTLSEI	279	287	BoLA-HD6	2.9
				BoLA-JSP.1	2.3
	IDLGPAISL	442	450	BoLA-D18.4	2.1
				BoLA-T2b	1.4
				BoLA-T2C	1.2
	IQALSYALG	227	235	BoLA-T2b	2.7
	IQVGSREYP	426	434	BoLA-D18.4	2.8
	KGIKARVTY	259	267	BoLA-D18.4	1.2
	KPDLTGTSK	532	540	BoLA-T2a	2.6
	LEKLDVGTN	450	458	BoLA-T2b	3
	LIANCASVL	382	390	BoLA-HD6	1.6
	LSKGNLIAN	377	385	BoLA-T2a	3
	LSYALGGDI	230	238	BoLA-HD6	1.8
				BoLA-JSP.1	1.2
	NALYPMSPL	342	350	BoLA-T2b	1.5
	PMSPLLQEC	346	354	BoLA-T2C	3
	RFILSKGNL	374	382	BoLA-T2b	2
	SIQALSYAL	226	234	BoLA-T2b	1
	SLMNSQAIE	136	144	BoLA-D18.4	3
				BoLA-T2C	2.5
	SQNALYPMS	340	348	BoLA-D18.4	2.7
				BoLA-HD6	1.7
				BoLA-D18.4	2.3
	STKSCARTL	358	366	BoLA-D18.4	3
				BoLA-HD6	2.7
				BoLA-JSP.1	1.6
				BoLA-T2b	2.9
				BoLA-T2C	2.7
	TGTSKSYVR	536	544	BoLA-T2a	2.9
	TKSCARTLV	359	367	BoLA-D18.4	2.3
	TLSEIKGVI	283	291	BoLA-D18.4	2.4
				BoLA-T2C	1.8
	YVATQGYLI	314	322	BoLA-HD6	2
				BoLA-T2C	2.8

N protein	ATLLKSLAL	2	10	BoLA-D18.4	1.6
				BoLA-JSP.1	1.3
				BoLA-T2a	2.6
				BoLA-T2b	1.9
	TLLKSLALF	3	11	BoLA-D18.4	2.6
				BoLA-HD6	3
				BoLA-T2C	1.3
	QQLGEVAPY	304	312	BoLA-HD6	2.2
				BoLA-JSP.1	3
				BoLA-T2a	1.4
				BoLA-T2b	2.9
	YFDPAYFRL	356	364	BoLA-JSP.1	1.3
				BoLA-T2b	2.4
				BoLA-T2C	1.5
	AYFRLGQEM	360	368	BoLA-HD6	1.6
				BoLA-JSP.1	2.1
				BoLA-T2C	2.9
	PRQAQVSFL	412	420	BoLA-JSP.1	1.7
				BoLA-T2b	2.2
				BoLA-T2C	2.6
	RSAEALFRL	490	498	BoLA-D18.4	1.2
				BoLA-HD6	1.2
				BoLA-T2a	1.9
				BoLA-T2b	2.9
				BoLA-T2C	2.7

## Data Availability

The [retrived strains, IEDB analysis methods] data used to support the findings of this study are included within the article.
